# Muscle Weakness and Walking Slowness for the Identification of Sarcopenia in the Older Adults from Northern Brazil: A Cross-Sectional Study

**DOI:** 10.3390/ijerph19159297

**Published:** 2022-07-29

**Authors:** Alex Barreto de Lima, Duarte Henrinques-Neto, Gustavo dos Santos Ribeiro, Elvio Rúbio Gouveia, Fátima Baptista

**Affiliations:** 1 CIPER, Faculdade de Motricidade Humana, Universidade de Lisboa, 1499-002 Cruz-Quebrada, Portugal; fbaptista@fmh.ulisboa.pt; 2 Course of Physical Education, State University of Amazonas, Manaus 69850-000, Brazil; 3 Sports and Health Department, Universidade Europeia, 1500-210 Lisboa, Portugal; duarteneto@campus.ul.pt; 4 Programa de Pós-Graduação em Ciências da Reabilitação, Universidade Federal de Ciências da Saúde de Porto Alegre (UFCSPA), Porto Alegre 90050-170, Brazil; gustavosr@ufcspa.edu.br; 5 Department of Physical Education and Sport, University of Madeira, 9000-072 Funchal, Portugal; erubiog@staff.uma.pt; 6 LARSYS, Interactive Technologies Institute, 9020-105 Funchal, Portugal

**Keywords:** gait speed, handgrip, sarcopenia, slowness, weakness

## Abstract

Background: This study aimed to analyze the prevalence of sarcopenia in elderly people from Northern Brazil according to muscle weakness or walking slowness. Methods: The sample consisted of 312 elderly people (72.6 ± 7.8 years). For walking slowness, a gait speed ≤ 0.8 m/s was used as a cut-off value, and for muscle weakness the following handgrip strength criteria were used for men and women, respectively: CI: <27.0/16.0 kg; CII: <35.5/20.0 kg; CIII: grip strength corrected for body mass index (BMI) < 1.05/0.79; CIV: grip strength corrected for total fat mass: <1.66/0.65; CV: grip strength corrected for body mass: <0.45/0.34. Results: Walking speed was reduced in 27.0% of women and 15.2% of men (*p* < 0.05). According to grip strength criteria, 28.5% of women and 30.4% of men (CI), 58.0% of women and 75.0% of men (CII), 66.0% of women and 39.3% of men (CIII), 28.8% of women and 19.6% of men (CIV), and 56.5% of women and 50.0% of men (CV) were identified as having sarcopenia. Conclusions: Walking slowness is more prevalent in women and muscle weakness is more prevalent in men in Northern Brazil. Walking slowness proved to be more concordant with muscle weakness in both sexes when the CI for handgrip strength was adopted.

## 1. Introduction

Muscle weakness is characterized by a lack of muscle strength and has diverse causes. Aging is the main cause of decreasing muscle strength, being identified as sarcopenia when a certain threshold is reached (according to the definitions proposed by different working groups) [1,2]. In addition to aging, disease, physical inactivity, sedentary behavior, and malnutrition are also relevant causes of sarcopenia [3]. Regardless of the cause, muscle strength is one of the key components when evaluating sarcopenia and is strongly associated with several negative outcomes in older adults [1]. Among those outcomes, limited mobility is commonly the first sign and predisposes older adults to functional disability, falls [1,2], fractures, increased risk of depression [4], hospitalizations [5], institutionalization, and premature death [6]. It is noteworthy that limited mobility seems to be even more important than multimorbidity to forecast mortality amongst older adults [7]. As a result of its predictive ability, assessment of muscle weakness and assessment of walking slowness (as a marker of limited mobility) are used for the identification of geriatric syndromes such as sarcopenia, frailty, and the risk of falling [1,8].

Some working groups focus the diagnosis of sarcopenia in assessing handgrip strength [1], while other groups recommend the assessment of either handgrip strength or gait speed as an alternative to each other [9]. Additionally, the cut-off values for identifying sarcopenia are different according to different working groups criteria, especially for handgrip strength, with a greater consensus for gait speed (≤0.8 m/s) [10,11,12]. This highlights the need for further studies [12].

Muscle weakness is usually assessed using an absolute muscle strength score [7,13,14,15] or by normalizing the absolute muscle strength to a body size variable [10,15]. Inaccuracy tends to increase in the first case, especially in older people with lower body mass and height [16]. Low absolute values may identify lighter and shorter body size older adults as having muscle weakness, even if they sustain their basic and instrumental activities of daily living [4]. On the other hand, the ratio standard procedure seems to overestimate the real strength of light/short older adults and underestimate it for tall/heavy ones [4], because of the nonlinear relationship between muscle strength and body-size variables [16].

Studies with particular body phenotypes, considering different regions of the globe, but in particular different regions of Brazil, e.g., interior of São Paulo [17], Nova Santa Rita [18], Macapá [19], Natal [20], and Manaus [21], contribute to the identification of vulnerable groups with greater urgency in the intervention. This mapping is essential to align public health policies with the needs of the elderly.

To contribute information about muscle weakness and walking slowness and sarcopenia prevalence from older people of Novo Aripuanã that is currently non-available, this study aimed: (1) to analyze the prevalence of sarcopenia in elderly people from Northern Brazil, according to the algorithms proposed by the European Working Group on Sarcopenia in Older People (EWGSOP) [1] and Sarcopenia Definitions and Outcomes Consortium (SDOC) [22] for muscle weakness and slow walking, and (2) to investigate the agreement of the prevalence between the slow walking and the different criteria of handgrip strength for sarcopenia. This information can be used to compare the prevalence of sarcopenia between sexes and age groups but also between national and international regions as a function of muscle weakness and slow walking [23], aiming at tailored public health interventions.

## 2. Methods

### 2.1. Sample and Study Design

This cross-sectional study was approved by the Ethics Committee of the State University of Amazonas (UEA) according to the Declaration of Helsinki and Resolution 466/12 of the National Health Council, making part of the research project: “Sarcopenic Syndrome—Physical Function, Phenotype and Quality of life in elderly with and without sedentary lifestyle” (CAAE 74055517.9.0000.5016/Referee 2.281.400).

**Figure 1:**
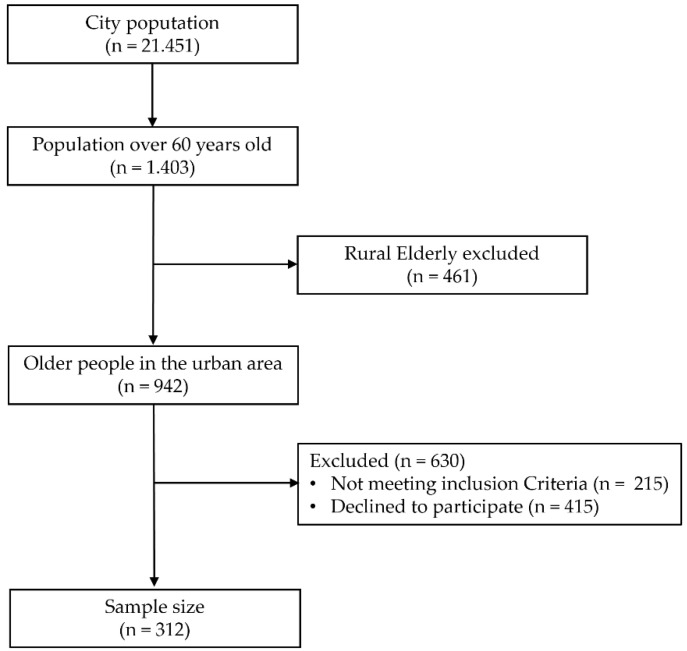
Flowchart diagram of the participant recruitment process.

The sample included 312 older adults from the community of Novo Aripuanã (Amazonas, Brazil). Participants were recruited in basic health units, parks, squares, churches, and other public places in the urban area of the city, in addition to invitations broadcast on local radio stations. Older adults living in rural areas were excluded from the study due to difficulties in accessing the evaluation site (distance and means of transportation needed) (Figure 1). After explanations about the procedures and risks of the study, all participants signed the informed consent form.

All assessments were performed out at UEA. The following criteria were considered for participant’s inclusion: (1) older aged 60 and over residing in the community; (2) be independent in carrying out activities of daily living; (3) moderate or high level of cognitive functioning; (4) no contraindications for physical exertion (stroke, neurological diseases, unstable chronic conditions); (5) without chest pain, and/or angina pectoris and limiting joint pain [24]. The cognitive level was evaluated with the Mini-Mental State Examination (MMSE) [25]. MMSE ≤ 15/30 points were used to exclude the participants of the study.

### 2.2. Instruments

#### 2.2.1. Socioeconomic Status

Participants reported sociodemographic data (age and education). The Brazilian Association of Research Companies [26] questionnaire was applied to evaluate socioeconomic status, which considers the possession of some consumer goods, educational level of the head of household, and access to public services.

#### 2.2.2. Muscle Weakness

Muscle strength was assessed using a handgrip dynamometer (Camry EH10; Sensun Weighing Apparatus Group Ltd., Shenzhen, China). Participants performed the handgrip strength test in a sitting position, with arms bent to 90 degrees in the elbow and shoulder joint. Both the left and right arms were measured twice. The results were recorded in kilograms (kg). The mean value of all measurements was used as the final score for each individual. Muscle weakness was identified using 5 criteria. The first criterion was according to the EWGSOP [1] and the remaining criteria were according to SDOC [22]. Criteria were the following: (I) <27.0 kg in men and <16.0 kg in women; (II) <35.5 kg in men and <20.0 kg in women; (III) grip over body mass index <1.05 for men and 0.79 for women; (IV) grip strength over total body fat <1.66 for men and <0.65 for women; (V) grip over bodyweight <0.45 for men and <0.34 for women.

#### 2.2.3. Slow Walking

To identify slow walking, we used the 4-m gait speed test (4-MGS) [27]. The 4-MGS speed is valid to assess gait speed, identify walking slowness, and the severity of sarcopenia [28,29,30]. Other distances (2.4 m to 15 m) are also used, but less frequently [31]. Subjects were asked to walk the course at their usual gait speed. Time taken to perform the walk was recorded, and the result was expressed as meters per second. If necessary, canes or walkers were permitted during this test. A gait speed ≤ 0.8 m/s was considered indicative of slow walking [2,32].

#### 2.2.4. Body Size and Composition

Body height, weight, fat-mass (FM), fat free mass (FFM), and muscle mass were assessed using anthropometric measures. All measurements followed the recommendations of the International Society for the Advancement of Kinanthropometry—ISAK [33]. Height and weight were measured using a mechanical scale (Welmy, São Paulo City, Brazil), girths of the relaxed arm, waist, abdomen, hip, and calf were measured with 2 m-metallic tape (Cescorf, Porto Alegre City, Brazil), and the skinfold triceps, calf, subscapular, and abdominal skinfolds using a skinfold caliper (Sanny, São Paulo City, Brazil). Muscle mass (MM) was estimated applying the equation proposed by Lee et al. [34]. Posteriorly, skeletal muscle mass index (SMMI) was obtained dividing muscle mass by height squared. Body fat was estimated using equations proposed by Williams et al. [35]. FM was estimated by multiplying the % body fat by weight (FM = weight x% body fat) and FFM was obtained by subtracting FM from weight (FFM = weight − FM). Lastly, the body mass index (BMI) was determined by dividing weight by height squared. Participants with a BMI ≥ 30 kg/m^2^ and a decreased grip strength according to criterion I were considered to have sarcopenic obesity [36].

### 2.3. Statistics

All analyses were performed using the Statistical Package for the Social Sciences (Version 24 for Windows; SPSS, Chicago, IL, USA). Data were stratified by sex and age range (60–69, 70–79, ≥80 years) and presented as mean ± standard deviation (SD) or percentage (%). Participants with muscle weakness and slow walking were identified according to the criteria described, and the respective prevalence (%) was calculated concerning the number of participants (total and by age group). Comparisons of prevalence between different age groups for the same sex and between the two sexes for the same age group were assessed using the Chi-Square Test. The proportions of limitations in handgrip strength and gait speed were also compared using the Cochran Q test. The level of agreement between participants with muscle weakness and slow walking was analyzed using the kappa statistic. The level of significance was set at *p* ≤ 0.05.

## 3. Results

A total of 312 older adults living in the community of Northern Brazil were evaluated (112 men and 200 women). Table 1 shows the educational level, body size, composition, and physical fitness of the participants. Participants were predominantly illiterate and female, with men presenting a higher level of academic education, greater muscle mass, gait speed, and handgrip strength (regardless of normalization for body composition) than women.

The prevalence of low muscle strength and low gait speed of the participants stratified according to the consensus definition of sarcopenia and age group is shown in Table 2 and Table 3. In women, differences in muscle weakness (criteria I and II) and walking slowness were observed. The ≥80-year-old group presented a higher prevalence compared to the younger groups. However, when muscle strength was adjusted for body composition, the prevalence of muscle weakness in women was similar in the three age groups. In men, there were differences in muscle weakness (criterion I, III, and V) and in walking slowness between age groups, from 70 years onwards compared to younger groups, except for handgrip strength criterion I, in which the differences were evidenced only in the ≥80-year-old group. Considering the gait speed, whose cutoff value for walking slowness is similar for both genders, women have a higher prevalence of walking slowness than men (*p* < 0.05) in different age groups, except for the 70–79-year-old group.

Table 4 presents the agreement between the diagnosis of walking slowness and muscle weakness assessed through different criteria (I–V). In men, there was agreement of the diagnosis of walking slowness with all muscle weakness criteria except criterion IV: KI = 0.287 ± 0.095; *p* = 0.001; KII = 0.113 ± 0.032; *p* = 0.010; KIII = 0.265 ± 0.082; *p* = 0.001; KIV = 0.041 ± 0.099; *p* = 0.661; kV = 0.196 ± 0.066; *p* = 0.004. In women, there was only agreement on the diagnosis of walking slowness with criteria I and II for muscle weakness, i.e., with the criteria not adjusted for body composition: KI = 0.265 ± 0.074; *p* = 0.0001; KII = 0.162 ± 0.055; *p* = 0.005; KIII = 0.076 ± 0.050; *p* = 0.143; KIV = 0.097 ± 0.073; *p* = 0.169; KV = 0.049 ± 0.058; *p* = 0.424. The level of agreement between variables was, however, fair, i.e., with a K value between 0.21–0.40.

**Figure 2:**
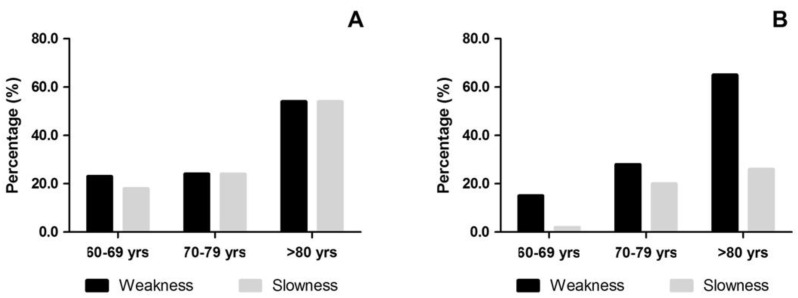
Comparison of the prevalence (%) of muscle weakness with gait slowness in women (**A**) and men (**B**); difference in prevalence in men aged 80 and over (*p* = 0.04).

Comparing the prevalence of slow walking with that of muscle weakness in criterion I, i.e., the criterion with the greatest agreement, differences were observed between these prevalence in men aged 80 years and over (Figure 2).

## 4. Discussion

This study aimed to describe the prevalence of sarcopenia in older adults from the Northern Brazil, according to the two main criteria based on muscle weakness and walking slowness and to investigate the concordance of the prevalence between these criteria. The results reveal a prevalence of ~20.0% in muscle weakness and walking slowness in women aged 60–79 years and more than 50.0% at 80 years and older, considering a handgrip strength < 16.0 kg as a cut-off value for the identification of sarcopenia (criterion I). Taking a handgrip strength < 20.0 kg as the cut-off value for the identification of sarcopenia in women (criterion II), the prevalence rises to ~53% between the ages of 60 and 79 years and to 81.0% at age 80 and over. In men, muscle weakness is more prevalent than slow walking, especially from the age of 80 where it reaches 65.0% (criterion I) or 87.0% (criterion II) against a prevalence of 26.1% for slow walking. Walking slowness was shown to be more concordant with muscular weakness when the cut-off value for handgrip strength was 16.0 kg for women and 27.0 kg for men (Criterion I).

The use of different cut-off values for muscle weakness in the present study is due to the lack of consensus that still exists regarding the assessment of sarcopenia [20]. The criteria adopted are normative (positioning of an individual in relation to a group) [1] or referred to functional capacity (mobility limitation) and risks (falls, hip fractures, mortality) [22]. However, in both cases, the establishment of cut-off values will depend not only on sexual dimorphism but possibly also on population polymorphism concerning body size [37,38,39,40].

The handgrip cut-offs have been widely used to diagnose sarcopenia in populations living in developed countries, where they were originally defined, so the use of such values in other populations may lead to inaccurate prevalence rates [41], particularly when the criteria is not adjusted for body dimensions. According the EWGSOP [1], the prevalence of muscle weakness in our study was ~30% considering the total sample in both men and women. This prevalence is higher than those observed in other regions of Brazil for the same muscle weakness criterion as in the Interior of São Paulo (17.5%) [17], Nova Santa Rita (23.7%) [18], Macapá (6.1%) [19], Natal (4.6%) [20], or Manaus [21].

As the comparison of the prevalence of sarcopenia between geographic regions can be affected by the composition of the groups in terms of sex and age, we proceeded with a more selective analysis: in addition to the average age in these studies being lower than that of our sample, possible differences in body height (not reported in some studies) may be the main reason for prevalence discrepancies. For example, the average handgrip strength for men and women aged 65 to 74 years in an international study was, respectively, 41.68 kg and 22.85 kg in Kingston (Canada), 34.09 kg and 20.78 kg in Tirana (Albania), 31.88 kg and 18.94 kg in Natal (Brazil) [42]. The corresponding values for our sample of Novo Aripuanã (Amazonas, Brazil), comprising only participants aged 65 to 74 years, was 31.62 kg and 20.24 kg for men and women, respectively, showing relatively close handgrip strength for similar body heights as is the case with our sample (men: 1.61 ± 0.07 m; women 1.51 ± 0.05 m) and the Natal sample in Brazil (men: 1.64 ± 0.07 m; women: 1.50 ± 0.05 m). Surprisingly, identical or higher values were evidenced for gait speed by our sub-sample aged 65 to 74 (men: 1.32 ± 0.36 m/s; women: 1.07 ± 0.32 m/s), having the same international study as a reference [42]. This observation may have to do with the usual physical activity of our sample whose livelihood comes from agriculture and fishing: 73.0% of women and 89.0% of men are considered active according to international recommendations [43].

Findings support the use of handgrip strength as a proxy for detecting slow walking speed (≤0.8 m/s) in community-dwelling older adults owing [44]. Several cutoff values for the handgrip have been proposed for this purpose [45,46,47,48,49,50]. We highlight the cutoff values of the group of Vasconcelos and colleagues (<25.8 kg for men; <17.4 kg for women) [48], but also of the group of Alley and colleagues (<26.0 kg for men; <16.0 kg for women) [50], as they are very similar to those observed in our sample to discriminate mobility limitation, namely 27.3 kg for men and 18.6 kg for women (data not shown; men: AUC 0.791, 95% CI: 0.689–0.892; Se 70.6%; Sp 74.7%; *p* < 0.001; women: AUC: 0.657, 95% CI: 0.565–0.749; Se 61.1%; Sp 63%; *p* = 0.001).

The latest SDOC panel confirmed the need to include muscle weakness and slowness in the definition/screening of sarcopenia because of its strong association with incidence of falls, hip fracture, and mobility limitation [10,22]. In addition, the low level of physical activity and muscle weakness related to age may affect the lower limbs, directly compromising the elderly autonomy [51]. Further research with larger samples and follow-up is needed to validate the cutoff values. It is also necessary to investigate the universality of cut-off values for muscle weakness and slow gait, considering not only physical impairment but also cognitive impairment [52].

Since sarcopenia has serious implications, early identification is an important task. Several sarcopenia evaluation tools have been proposed and it is necessary to investigate its validity in different population groups due, at least, to differences in body dimensions. For example, the population of Novo Aripuanã in Northern Brazil has lower body dimensions than other populations in southern and southeastern Brazil, where the prevalence of sarcopenia has been characterized [17,18,41,53].

In recent years, sarcopenia has been discussed by two large working groups: the EWGSOP2 [1] and the SDOC [22]. Both agree with the general concept that involves impairment of function (muscle weakness and slowness). However, there is divergence concerning the third component of screening: structural damage (low muscle quantity/quality). While the EWGSOP2 recommends that low muscle mass be the confirmation criterion for sarcopenia, the SDOC does not consider muscle mass (evaluated by DXA or BIA) in its guidelines, as it has not been associated with adverse outcomes in longitudinal studies and large clinical trials [22]. Thus, the SDOC proposes the interpretation of the handgrip strength with or without adjustment for body mass or body mass index, body fat, or arm muscle mass [22]. However, we did not find handgrip strength studies with this type of adjustment for comparative purposes.

The suggested use of muscle weakness or walking slowness for the identification of sarcopenia [22,50,54] raises the question of the possibility of agreement between the two for diagnosis, although the correlation appears to be weak between the values of these two criteria [55].

## 5. Study Limitations and Strengths

We acknowledged some limitations of the present study that must be considered when interpreting the results. First, the cross-sectional design is inadequate to capture the temporal relations that occur throughout life, and this approach precludes inference of causality between muscle weakness or walking slowness and sarcopenia. However, besides this being a descriptive study, we considered the algorithms proposed by EWGSOP2 [1] and SDOC [22] for muscle weakness and slow walking in the calculations of sarcopenia. Second, the sample did not include older people living in rural areas and only included participants that could walk without assistance or aid of other people where the assessments were conducted. The generalizability of our findings to less-mobile populations of Novo Aripuanã and people who live in rural areas is not possible. Third, the heterogeneity among the participants of this study can introduce bias in the identification of sarcopenia due to different physical phenotypes (larger dimensions), favoring the increase in the prevalence of muscle weakness and walking slowness in our sample.

However, when women’s handgrip is adjusted for body size (BMI or body mass), the prevalence of muscle weakness remains or increases, while in men, it remains or decreases. Finally, the prevalence of muscle weakness is also affected by morbidity, information which was not properly collected in this study, constituting a limitation, especially at the level of comparison between geographic regions. Nonetheless, the strongest point is the analysis of the agreement between muscle weakness and walking slowness for the identification of sarcopenia in this population of the northern region of Brazil. We acknowledged that further research with larger samples and follow-up is necessary to validate the cutoff values of muscle weakness and walking slowness in intrinsic capacity and adverse events such as falls, hospitalization, and mortality.

## 6. Conclusions

In conclusion, walking slowness was more prevalent in women than in older men from the north of Brazil, while muscle weakness was more prevalent in men. The prevalence of muscle weakness seems to be higher in this elderly population than in other regions of Brazil or internationally. Despite a weak level of agreement, the walking slowness was more in agreement with muscle weakness in both sexes when the criterion was that of the EWGSOP2.

## Figures and Tables

**Table 1:** Characteristics of the participants.

Variables	Overall	Men	Women	*p*-Value
Sample size, *n*	312	112	200	<0.001
Age, years	72.6 ± 7.8	73.1 ± 7.3	72.4 ± 8.1	0.232
Educational level				
Non-literate, *n* (%)	176 (56.4)	63 (56.3)	113 (56.5)	1.000
Elementary school, *n* (%)	83 (26.6)	29 (25.9)	54 (27.0)	0.894
High school, *n* (%)	28 (9.0)	6 (5.4)	22 (11.0)	0.103
Graduate or above, *n* (%)	25 (8.0)	14 (12.5)	11 (5.5)	0.048
Mini mental, score	21.2 ± 5.1	21.8 ± 4.6	20.8 ± 5.3	0.124
Body size and composition				
Height, cm	153.7 ± 8.2	160.0 ± 8.3	150.1 ± 5.7	<0.001
Weight, kg	63.7 ± 12.7	69.3 ± 11.6	60.5 ± 12.2	<0.001
BMI, kg/m²	26.9 ± 4.7	27.1 ± 4.6	26.8 ± 4.7	0.588
Muscle mass, kg	19.9 ± 4.6	23.7 ± 3.6	17.7 ± 3.6	<0.001
SMMI, kg/m²	8.3 ± 1.5	9.2 ± 1.2	7.8 ± 1.4	<0.001
Sarcopenic obesity, *n* (%)	57 (18.3)	22 (19.6)	35 (17.5)	0.253
Physical performance				
Gait speed, m/s	1.09 ± 0.36	1.20 ± 0.35	1.03 ± 0.35	<0.001
Muscle strength				
Handgrip strength, kg	23.7 ± 9.2	31.4 ± 8.9	19.3 ± 5.9	<0.001
Handgrip/body mass, kg/kg	0.37 ± 0.13	0.46 ± 0.12	0.33 ± 0.10	<0.001
Handgrip/BMI, kg/kg.m^−2^	0.89 ± 0.35	1.18 ± 0.34	0.74 ± 0.23	<0.001
Handgrip/body fat, kg/kg	1.41 ± 0.88	2.07 ± 1.08	1.04 ± 0.43	<0.001

BMI: body mass index. SMMI: skeletal muscle mass index.

**Table 2:** Prevalence (%) of low muscle strength and low gait speed in elderly of Northern Brazil according to consensus definition of sarcopenia and age group—WOMEN.

Age Group (Years) Participants (*n*)	Overall	60–69	70–79	≥80	*p*-Value
	200	93	70	37	
EWGSOP (2019)					
Low muscle strength	57 (29.0)	20 (21.5) _a_	17 (24.3) _a_	20 (54.1) _b_	0.001
SDOC (2020)					
Low muscle strength	116 (58.0)	49 (52.7) _a_	37 (52.9) _a_	30 (81.1) _b_	0.007
Low muscle strength/BMI	132 (66.0)	58 (62.4) _a_	45 (64.3) _a_	29 (78.4) _a_	0.205
Low muscle strength/FM	56 (28.0)	23 (24.7) _a_	21 (30.0) _a_	12 (32.4) _a_	0.609
Low muscle strength/BM	113 (56.5)	52 (55.9) _a_	39 (55.7) _a_	22 (59.5) _a_	0.922
Low gait speed	54 (27.0)	17 (18.3) _a_	17 (24.3) _a_	20 (54.1) _b_	≤0.001

EWGSOP, European Working Group on Sarcopenia in Older People; SDOC, Sarcopenia Definition and Outcomes Consortium; BMI, body mass index; FM, fat mass; BM, body mass. Values expressed as *n* (%). a and b in subscript indicate the existence (or not) of significant differences between the proportions of the groups according to the Chi-square test; equal letters indicate no differences (*p* ≤ 0.05).

**Table 3:** Prevalence (%) of low muscle strength and low gait speed in elderly of Northern Brazil according to consensus definition of sarcopenia and age group—MEN.

Age Group (Years) Participants	Overall	60–69	70–79	≥80	*p*-Value
	112	39	50	23	
EWGSOP (2019)					
Low muscle strength	34 (30.4)	6 (15.4) _a_	13 (26.0) _a_	15 (65.2) _b_	≤0.001
SDOC (2020)					
Low muscle strength	84 (75.0)	26 (66.7) _a_	38 (76.0) _a_	20 (87.0) _a_	0.199
Low muscle strength/BMI	44 (39.3)	9 (23.1) _a_	22 (44.0) _a,b_	13 (56.5) _b_	0.022
Low muscle strength/FM	22 (19.6)	9 (23.1) _a_	7 (14.0) _a_	6 (26.1) _a_	0.386
Low muscle strength/BM	56 (50.0)	12 (30.8) _a_	29 (58.0) _b_	15 (65.2) _b_	0.010
Low gait speed	17 (15.2)	1 (2.6) _a_	10 (20.0) _b_	6 (26.1) _b_	0.020

EWGSOP, European Working Group on Sarcopenia in Older People; SDOC, Sarcopenia Definition and Outcomes Consortium; BMI, body mass index; FM, fat mass; BM, body mass. Values expressed as *n* (%). a and b in subscript indicate the existence (or not) of significant differences between the proportions of the groups according to the Chi-square test; equal letters indicate no differences (*p* ≤ 0.05).

**Table 4:** Cross classification analysis between positive (+) cases for muscle weakness and for walking slowness by sex.

		Gait Slowness					
		Men			Women		
Muscle Weakness Criteria (C)		−Cases	+Cases	Total	−Cases	+Cases	Total
CI_ Handgrip Strength	−cases	64.3%	5.4%	69.6%	57.5%	14.0%	71.5%
	+cases	20.5%	9.8%	30.4%	15.5%	13.0%	28.5%
CII_ Handgrip Strength	−cases	25.0%	0.0%	25.0%	35.0%	7.0%	42.0%
	+cases	59.8%	15.2%	75.0%	38.0%	20.0%	58.0%
CIII_ Handgrip/BMI	−cases	57.1%	3.6%	60.7%	27.0%	7.0%	34.0%
	+cases	27.7%	11.6%	39.3%	46.0%	20.0%	66.0%
CIV_ Handgrip/FM	−cases	68.8%	11.6%	80.4%	54.5%	17.5%	72.0%
	+cases	16.1%	3.6%	19.6%	18.5%	9.5%	28.0%
CV_ Handgrip/BM	−cases	47.3%	2,7%	50.0%	33.0%	10.5%	43.5%
	+cases	37.5%	12.5%	50.0%	40.0%	16.5%	56.5%

CI_Hanggrip strength: <27.0 kg for men and <16.0 kg for women; CII_Handgrip strength: <35.5 kg for men and < 20.0 kg for women; BMI: body mass index; FM: Fat Mass; BM: body mass.

## Data Availability

The data presented in this study are available on request from the corresponding author. The data are not publicly available due to belonging to a database of a Ph.D. thesis in progress.
